# Diagnosis and treatment of carcinosarcoma of the renal pelvis: A case report

**DOI:** 10.3892/ol.2014.2145

**Published:** 2014-05-13

**Authors:** BIAO DONG, JIAN-JIAN ZHANG, CHAO CHEN, YUAN-TAO WANG, XIU-YU ZHAI, YAO-WEN FU, HONG-LAN ZHOU

**Affiliations:** 1 Department of Urology, First Hospital of Jilin University, Jilin, Changchun 130021, P.R. China; 2 Department of Hepatic Surgery, Renji Hospital, Shanghai Jiaotong University School of Medicine, Shanghai 200127, P.R. China; 3 Department of Critical Care Medicine, Henan Province People’s Hospital, Zhengzhou, Henan 450003, P.R. China

**Keywords:** carcinosarcoma, renal pelvis, squamous cell carcinoma, fibrosarcoma

## Abstract

Carcinosarcoma is a rare type of renal pelvis malignancy, the diagnosis of which requires the presence of malignant epithelial and mesenchymal components. The prognosis of this disease is extremely poor due to its rapid progression and widespread metastases. The present study describes a case of carcinosarcoma involving the right renal pelvis in a 73-year-old female who presented with intermittent hematuria and right-flank pain that had persisted for one month. Computed tomography revealed a 2.4×2.5 cm mass in the right renal pelvis, which was diagnosed as a right renal pelvic tumor. Laparoscopic radical resection of the right kidney and ureter was performed. Following surgery, immunohistochemical analysis showed positive reactions for epithelial and mesenchymal markers. Based on these findings, the patient was diagnosed with carcinosarcoma. Thus, immunohistochemical analysis is a critical method for the accurate diagnosis of carcinosarcoma.

## Introduction

Carcinosarcoma is a rare neoplasm that shows an admixture of epithelial and mesenchymal components (1,2). The carcinomatous component of carcinosarcoma may be represented by varying forms, including transitional cell carcinoma, adenocarcinoma or squamous cell carcinoma (SCC). The sarcomatous portion shows specific features of mesenchymal differentiation, with elements that include chondrosarcoma, osteosarcoma, rhabdomyosarcoma, liposarcoma and fibrosarcoma (3–6). Carcinosarcoma is often localized in a wide variety of organs, including the uterus, breast, esophagus, larynx, lungs, urinary bladder, prostate and oviducts, with a variable frequency; however, localization in the renal pelvis is rare (7). The present study reports a case of carcinosarcoma of the renal pelvis in a 73-year-old female, which consisted of SCC and fibrosarcoma components, and discusses the diagnosis and treatment of such tumors. Patient provided written informed consent.

## Case report

### Patient presentation

A 73-year-old female was admitted to the Department of Urology, First Hospital of Jilin University (Jilin, China) presenting with intermittent hematuria and right-flank pain for one month. The patient had a history of hysterectomy for uterine fibroids 10 years previously. The patient had no other voiding complaints or any significant urological history. The patient also denied past tobacco use or analgesic abuse, and the results of the physical examination were normal, except for mild percussion pain in the right kidney area. Urinalysis revealed increased numbers of red blood cells, leukocytes and suspicious malignant cells. Furthermore, protein was found to be excreted in the urine (score, 1+) and the analysis of the blood biochemistry revealed an elevated erythrocyte sedimentation rate (90 ml/h).

### Tumor imaging and resection

**Figure 1:**
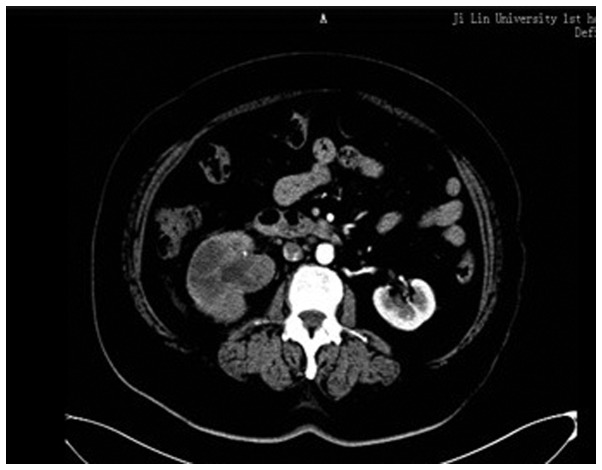
CT scan showing a well-defined 2–3-cm mass invading the right renal pelvis. The mass shows poor contrast enhancement.

Abdominal ultrasonography showed a solid, relatively well-demarcated tumor, 3.0×3.1 cm, occupying the right renal pelvis. Computed tomography (CT) showed a 2.4×2.5-cm heterogeneous and poorly-enhanced mass in the right renal pelvis (Fig. 1). The CT findings also revealed a thickened ureter wall, with irregular contrast enhancement. Cystoscopy showed no abnormalities in the urinary bladder. Retrograde pyelography could not be performed due to ureteral catheter obstruction. Further clinical analyses revealed no metastasis to other organs. Based on the clinical and radiological findings, a laparoscopic radical resection of the right kidney and an open ureterectomy were performed to remove the tumors.

### Macroscopic and histological tumor analysis

**Figure 2:**
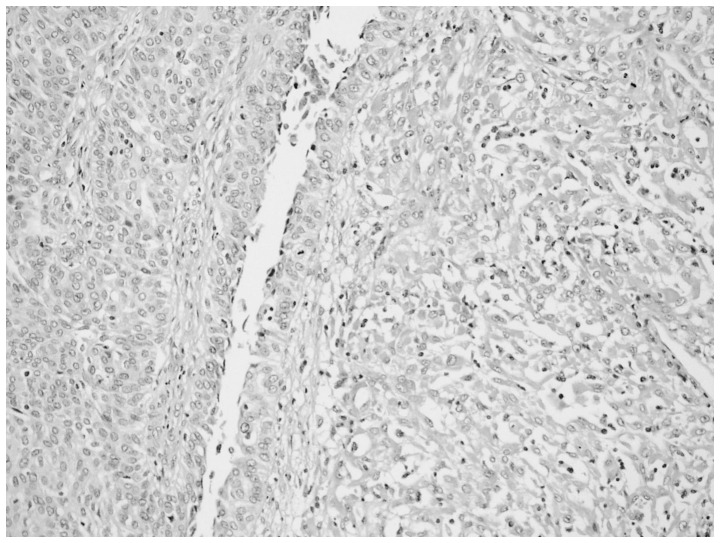
Biphasic tumor composed of an epithelial component and a mesenchmal component. Frequent mitotic figures are observable among the tumor cells. Stain, hematoxylin and eosin; magnification, ×20.

**Figure 3:**
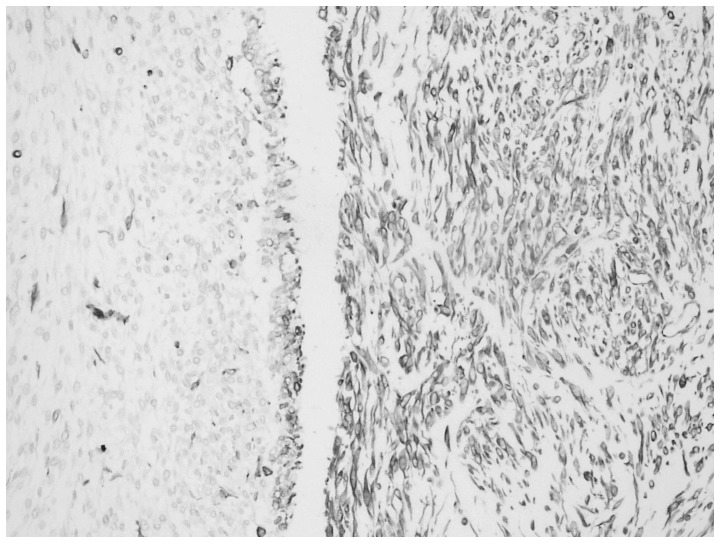
Immunohistochemical staining showing immunoreactivity for cytokeratin in the epithelioid cells of the carcinosarcoma. Magnification, ×20.

**Figure 4:**
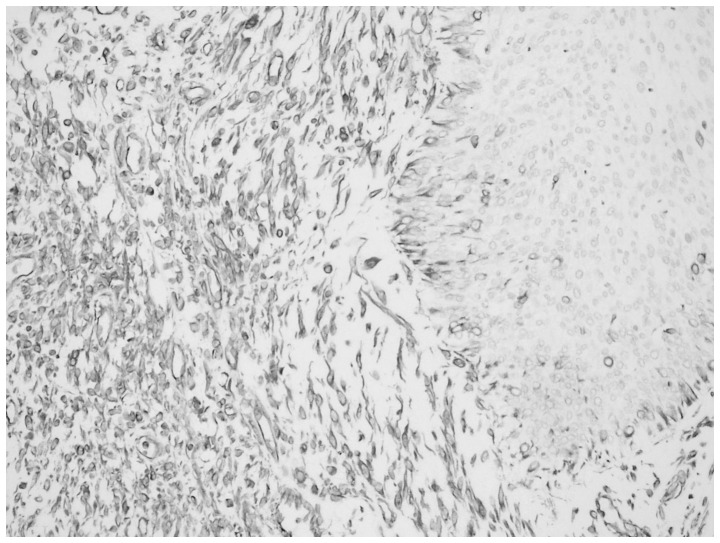
Immunohistochemical staining showing immunoreactivity for vimentin in the spindle cells of the carcinosarcoma. Magnification, ×20.

Macroscopic examination of the 13×8×6-cm nephrectomy specimen revealed a 8×5×4-cm tumoral mass in the renal pelvis. The cut section of the mass was gray-white in color, with a hard consistency. Areas of extensive necrosis were also present. The tumor had invaded the full-thickness of the renal pelvic wall and peripelvic adipose tissue. Furthermore, invasion into the renal parenchyma was observed. Histological examination of the tumor showed a malignant neoplasm comprising of epithelial and mesenchymal components, which were largely separated from each other (Fig. 2). However, in certain areas, the epithelial component blended into the sarcomatous component, generating a histological transition between the two. The sarcomatous areas primarily consisted of spindle cells, which were full of eosinophilic cytoplasm. Immunohistochemical staining was performed using a panel of markers, including cytokeratin, vimentin, Ki-67 antigen and p53. The epithelial portion of the tumor was found to stain positively for cytokeratin (Fig. 3) and the sarcomatoid spindle cells were observed to stain positively for vimentin, but negatively for cytokeratin (Fig. 4). The tumor cells in the epithelial and sarcomatous components were also found to express p53 protein in the nuclei. The expression level of p53 was >10%. Furthermore, the Ki-67 labeling indices were >20% in all of the tumor cells. Due to the advanced age of the patient, chemotherapy and radiotherapy were not administered. The patient was discharged six days after surgery and no recurrence was observed after eight months.

## Discussion

Carcinosarcoma is a rare, malignant neoplasm that shows histological evidence of intimately mixed epithelial and mesenchymal elements (3). The histogenesis of carcinosarcomas remains controversial and there are two predominant theories. Völker *et al* (8) proposed that carcinosarcomas may originate from a common pluripotent progenitor cell that is capable of undergoing epithelial and mesenchymal differentiation. Perret *et al* (9) proposed that certain carcinosarcomas should be regarded as a variant of sarcomatoid carcinoma (metaplastic carcinoma) that shows prominent heterologous differentiation.

Carcinosarcoma has been described in various organs, including the uterus, breast, stomach, lung, salivary glands, thyroid gland and gallbladder. However, carcinosarcoma of the urogenital organs has rarely been reported (1–4,6,10–15).

Carcinosarcoma of the renal pelvis has been shown to be aggressive and often has a poor prognosis (16–18), thus it is important to detect and diagnose this disease early. Microscopically, the carcinomatous component is primarily composed of transitional cell carcinoma and the sarcomatous component is predominantly composed of spindle and/or pleomorphic tumor giant cells (11). In the present case, light microscopy revealed epithelial and sarcomatous components, which were largely separated from each other. However, in certain areas, the epithelial component blended into the sarcomatous component, generating a histological transition between the two.

Due to the similar microscopic appearance of carcinosarcomas and sarcomatoid carcinomas, immunohistochemistry may be a useful diagnostic adjunct for differentiating between these tumors. In the present case, antigenic determinants that were specific for epithelial cells, such as cytokeratin, were identified. Furthermore, the sarcomatous component was characterized by strong staining for vimentin. In addition, the lack of expression of keratin markers in the mesenchymal component further confirmed the diagnosis of carcinosarcoma.

No clinical trials have been specifically designed for carcinosarcoma, thus an optimal treatment strategy has yet to be established. At present, radical resection is the only curative treatment. Nephrectomy, radiation therapy and chemotherapy have been used alone or in combination (1,19). In the present case, due to the age of the patient and the history of previous pelvic surgery, a laparoscopic radical resection of the right kidney and ureter was performed. Previous studies have reported very poor prognoses for carcinosarcoma, with a median cancer-specific survival time of approximately one year (6,9,20). One half of patients are reported to succumb within approximately one year of diagnosis. Chen *et al* (12) reported the longest survival period of two years. Cancer-specific survival times have been found to be significantly improved for patients who undergo radical resection rather than chemotherapy or radiation therapy.

In conclusion, carcinosarcoma of the renal pelvis is a rare, aggressive tumor, with a low survival rate. Although rare, carcinosarcoma should be included in the differential diagnosis. Further investigations into the natural history and prognostic factors of this disease and specific guidelines regarding therapeutic approaches for this tumor are urgently required.
